# Advancing the Frontiers: Contemporary Achievements and Future Directions in Density Functional Theory Calculations

**DOI:** 10.3390/molecules31040676

**Published:** 2026-02-15

**Authors:** Aleksandr S. Kazachenko, Noureddine Issaoui

**Affiliations:** 1 Institute of Chemical Technologies, Reshetnev Siberian State University of Science and Technology, Markovskogo St. 57, 660049 Krasnoyarsk, Russia; 2 Institute of Chemistry and Chemical Technology, Federal Research Center “Krasnoyarsk Scientific Center” Siberian Branch, Russian Academy of Sciences, Akademgorodok St. 50/24, 660036 Krasnoyarsk, Russia; 3 School of Non-Ferrous Metals and and Material Science, Siberian Federal University, Pr. Svobodnyy 79, 660041 Krasnoyarsk, Russia; 4 Voino-Yasenetsky Krasnoyarsk State Medical University of the Ministry of Healthcare of the Russian Federation, St. Partizan Zheleznyak, Bld. 1, 660022 Krasnoyarsk, Russia; 5 Laboratory of Quantum and Statistical Physics (LR18ES18), Faculty of Sciences, University of Monastir, Avenue de l’Environnement, Monastir 5019, Tunisia; issaoui_noureddine@yahoo.fr

Density Functional Theory (DFT) has been a cornerstone of computational chemistry and physics for several decades [1,2,3,4]. Its unique combination of relative computational efficiency and reasonable accuracy has made it an indispensable tool for probing the atomic and electronic structure of materials [5,6,7], molecules [8,9,10], and surfaces [11,12,13,14]. The collection of articles presented in this Special Issue vividly illustrate the incredible breadth of DFT’s applications—from geochemistry and catalysis to pharmaceuticals and materials science. This editorial aims not only to summarize the key trends showcased in the issue but also to outline the new frontiers contemporary DFT is approaching and to discuss the persistent challenges that accompany its evolution.

The diversity of applications showcased here underscores DFT’s remarkable versatility in bridging scales from the atom to complex macroscopic systems. Modern research demonstrates that the scope of DFT is continuously expanding to encompass increasingly complex phenomena. In the field of reactivity and catalysis, a classical domain for DFT, studies on the thermal decomposition of triazolones and their thione analogs [15], and the hydrogenation of CO_2_ on doped copper surfaces [16], exemplify how DFT elucidates not only the sequence of elementary steps, but also enables the rational selection of functionals for different compound classes. This includes accounting for the higher polarizability of sulfur-containing systems or better modeling dispersion interactions in those that are oxygen-containing [17,18,19]. Furthermore, DFT identifies key activity descriptors, such as the dramatic reduction in oxygen vacancy formation energy in samaria-doped ceria, a process central to its catalytic function [20]. The method successfully transcends molecular systems to tackle complex materials and nanostructures. Investigations into the diffusion of ionic liquids within metal–organic frameworks (MOFs) [21,22,23], the stability of pharmaceutical hydrate forms like thiamine hydrochloride [24], and the interaction of boron oxide nanoflakes with the antiviral drug favipiravir [25] demonstrate how periodic calculations and cluster models provide atomistic insight into the properties of composites, polymorphs, and nanocarriers. This capability extends to fundamental questions in geochemistry and environmental science. The study of protolysis reactions on pyrophyllite surfaces [26] and the photolysis of β-butyrolactone in the atmosphere [27] are prime examples of DFT offering an atom-scale understanding of mineral dissolution and organic pollutant degradation processes that are often inaccessible through experiment alone [28,29]. Moreover, DFT is making significant inroads into biochemistry and pharmacology. The use of conceptual DFT (CDFT) and pharmacokinetic property assessment for screening saxitoxin derivatives as potential pharmaceutical candidates [30] represents an increasingly promising direction that marries quantum–chemical calculations with bioinformatic approaches, opening new avenues for drug discovery [31,32].

A critical dimension reinforced by several studies in this collection is the synergy between DFT calculations and experimental validation, which is paramount for transforming theoretical predictions into reliable knowledge [33,34]. The work on the electronic spectroscopy of β-butyrolactone [27] stands as a direct example, where time-dependent DFT (TD-DFT) calculations are employed to assign features in a novel vacuum ultraviolet absorption spectrum. Similarly, the comprehensive analysis of the histaminium bis(trioxonitrate) compound [35] and N-Butyl-1H-benzimidazole [36] meticulously compares calculated structural parameters, vibrational spectra, and UV–Vis transitions with experimental data from X-ray diffraction, FTIR, and spectrofluorimetry. This iterative dialog between computation and experiment not only validates the theoretical models and chosen levels of theory, but also provides a deeper, mechanistic interpretation of the raw experimental data [37,38,39]. In materials science, this synergy is equally vital. The DFT study of gold–ligand interactions [40,41,42] provides the electronic structure rationale for the empirically observed stability series of gold complexes, thereby informing the design of new leaching agents. This back-and-forth is the engine of modern molecular and materials design, where DFT screens promising candidates and experiments confirm their real-world properties, closing the discovery loop [43,44,45]. The detailed study of binary systems, such as the ammonium sulfamate–urea system [46] and the sulfamic acid–urea system [47], further highlights how combined theoretical and experimental techniques (FTIR, XRD, TGA/DSC, DFT, QTAIM) are essential for understanding the thermal stability and physicochemical behavior of complex mixtures.

However, progress in these exciting applications inevitably encounters methodological limitations, and simultaneously stimulates the development of novel approaches, pointing toward new horizons for the theory [48,49]. One of the central themes, highlighted in the work on triazolone decomposition [15], is the critical dependence of results, particularly activation energies, on the choice of the exchange–correlation functional. The future lies with the systematic use of modern, robust, hybrid, and dispersion-corrected functionals (e.g., those from the r^2^SCAN or ωB97 families) and, increasingly [50,51,52], with the integration of machine learning (ML) [53,54,55,56,57,58,59]. ML is poised to revolutionize DFT by creating accurate and transferable interatomic potentials, predicting material properties across vast chemical spaces, and even guiding the development of new functionals themselves, forming a new paradigm for computational discovery [60,61,62,63]. Another frontier involves the modeling of “soft matter” and dynamic processes in condensed phases. The investigation of ionic liquid diffusion in flexible ZIF-8 [21] underscores the complexity of simulating dynamics within pliable porous frameworks. Advances in DFT-based molecular dynamics coupled with enhanced sampling techniques are crucial for studying rare events, phase transitions, and solvation effects, thereby bridging the gap between static zero-Kelvin calculations and real-world experimental conditions [64,65,66,67,68,69]. Furthermore, the accurate description of excited states and non-adiabatic processes remains a vibrant area of development. The work on β-butyrolactone using TD-DFT [27] exemplifies the growing interest in photochemistry and excited-state dynamics. The development of methods for accurately describing conical intersections, charge transfer states, and multielectron excitations—often using higher-level wavefunction methods as benchmarks for TD-DFT—is critical for progress in photovoltaics, optoelectronics, and spectroscopy [70,71,72,73,74,75]. The importance of including exact Hartree–Fock exchange for correctly describing vibronic structure in electronic spectra, as discussed in foundational studies [76,77,78], continues to influence modern functional development for excited-state properties.

Looking ahead, the next wave of DFT applications will likely be dominated by the quest to understand and design functional materials under operating conditions and to tackle ever-larger and more disordered systems [79,80]. This includes the explicit simulation of electrochemical interfaces [81] for green energy technologies, where the interplay of electrode potential, solvation [82,83], and reaction kinetics poses a formidable challenge [84,85,86]. A pertinent example is the DFT-driven discovery of hollow spherical beryllium clusters as ultra-high-capacity, potentially reversible hydrogen storage materials, highlighting the theory’s direct role in advancing clean energy solutions [87,88,89,90,91]. Furthermore, the prediction of novel quantum materials with correlated electronic states or topological properties pushes standard DFT formulations to their limits, necessitating close collaboration with many-body perturbation theory (GW) [92,93] or dynamical mean-field theory (DMFT) [94,95,96]. The study of two-dimensional ice I and its anti-icing potential [97] hints at the fascinating interface of low-dimensional materials and phase behavior, a vast playground for DFT exploration. In the life sciences, the integration of DFT with classical molecular mechanics in QM/MM schemes will become more routine, enabling the study of enzyme catalysis and metalloprotein function with quantum-mechanical accuracy for the active site [98,99,100]. The ultimate challenge lies in moving from mere understanding to true inverse design: using algorithms to navigate the immense space of possible molecules and materials towards those that meet a specific set of target properties, with DFT serving as the essential scoring function [101]. The re-investigation of fundamental principles like Sabatier’s principle using free energy diagrams derived from DFT, especially in electrocatalysis [84], underscores the theory’s role in refining our most basic conceptual frameworks for activity prediction [102,103,104,105].

The future trajectory of DFT lies in its deep integration with complementary methods and experimental data. It is becoming the core engine of multiscale modeling frameworks, where quantum–chemical insights provide parameters for mesoscopic and macroscopic simulations. An important trend is the creation of “digital twins” for chemical processes—comprehensive computational models that integrate DFT-derived reaction pathways, microkinetic modeling, and real-time data analysis. DFT’s role is expanding from a tool for explanation to a platform for prediction and autonomous discovery. The articles compiled in this Special Issue provide compelling evidence that DFT remains a vital and dynamically evolving field. It continues to address fundamental questions in chemical physics while simultaneously becoming a key enabling technology for solving applied challenges in catalysis, materials design, drug development, and environmental science. By overcoming current methodological constraints through the development of next-generation functionals, integration with machine learning, and robust multiscale modeling, DFT is paving the way for a new era of predictive computational science. In this era, in silico modeling will be an indispensable and integral part of the discovery and innovation cycle, seamlessly connecting the quantum world to macroscopic properties and functions.
